# Permanent Severe Visual Field Defect Following Pre-eclampsia and Multiple Ophthalmological Pathologies: A Case Report

**DOI:** 10.7759/cureus.63052

**Published:** 2024-06-24

**Authors:** Nasim Abdouli, Thomas Haulot, Sayeh Pourjavan

**Affiliations:** 1 Ophthalmology, Cliniques Universitaires Saint-Luc, Brussels, BEL

**Keywords:** systemic lupus erythema, optic nerve ischemia, pre-eclampsia, visual field defect, glaucoma suspect, high myopia

## Abstract

This clinical report discusses the interplay of various pathologies that may present similar clinical manifestations, with uncertainty about the distinct impact of each one of them.

The patient is a 43-year-old young Asian female with no known medical conditions. She was 33 weeks pregnant when she was admitted for an urgent c-section because of preeclampsia with HELLP syndrome. While hospitalized, she complained about the visual field’s loss. A comprehensive ophthalmological examination revealed a severe concentric visual field defect along with well-reduced visual acuity and impaired color vision. Her OCT revealed a bilateral serous macular detachment related to pre-eclampsia. A brain MRI revealed a microstroke at the temporo-parieto-occipital junction (TPO), although it did not fully account for the severity of the visual field deficit. Despite the macular pathology being resolved, the visual field remained deeply impacted. A thorough and complete investigation yielded negative results, leaving the cause of the patient’s deficit unknown. The patient likely had a normal pressure glaucoma. Additionally, multifactorial bilateral microvascular ischemic neuropathy (caused especially by high myopia) has significantly affected her visual field. Furthermore, it is also probable that the patient had genetic neuropathy. Initial genetic testing was negative; however, due to the high suspicion of a genetic component, a retest was conducted, and the results were not conclusive.

This case represents a highly unusual case of a profoundly affected visual field with no apparent identified cause. This is a notable example of the potential interaction of various local and systemic pathologies that can manifest with similar clinical presentations.

## Introduction

Pre-eclampsia is a systemic pregnancy pathology that is related to hypertension and multiorgan dysfunction. It is estimated that this pathology affects 4-5% of pregnancies worldwide [1]. Visual field defects represent a crucial clinical manifestation associated with various ophthalmological and non-ophthalmological pathologies. The interpretation of these deficits plays a pivotal role in both diagnosis and disease monitoring. Automatic perimetry is the preferred method for assessing visual field defects. Common visual field alterations during pregnancy include bitemporal deficits, central dark spots [2], and enlarged blind spot patterns. Multiple ophthalmic pathologies could lead to those defects, but they are usually quickly reversible.

While many visual field issues encountered in clinical practice can be attributed to common pathologies, the challenge arises when a severe visual field defect is present and persists without an apparent cause. This case report discusses the case of a severe concentric and permanent visual field following preeclampsia.

## Case presentation

A 43-year-old Asian female, with no previous known medical condition, was pregnant with a twin pregnancy (of 33 weeks and 3 days, multiple previous ineffective in vitro fertilization). She sought emergency care due to significant left abdominal pain. In the emergency department workup, the patient had acute stage II arterial hypertension (160/100 mmHg) as well as proteinuria (4x). Blood analysis revealed elevated CRP (25 mg/l), hemoconcentration (17 g/dl), increased leucocytes (16 026/ml), low platelets (101 000/ml), increased creatinine (1,19 mg/dl), and cellular and hepatic lysis (LDH 425, GOT 39 & GPT 20). Fortunately, the ultrasound did not show fetal distress.

The diagnosis of pre-eclampsia with acute renal insufficiency and HELLP syndrome was established. This diagnosis led to an urgent C-section that resulted in the birth of two baby girls.

Shortly after the c-section, the patient started to complain about the loss of her visual field in the right eye. An immediate ophthalmologic examination was requested by the obstetrician, and that was our first encounter with the patient in the ophthalmology department.

**Figure 1:**
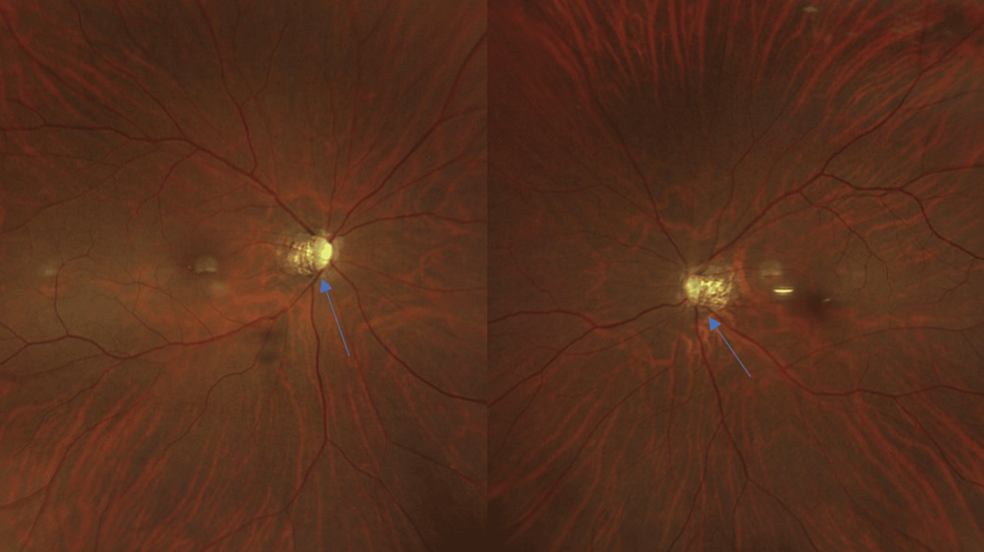
Fundus photography Note the tilted discs

**Figure 2:**
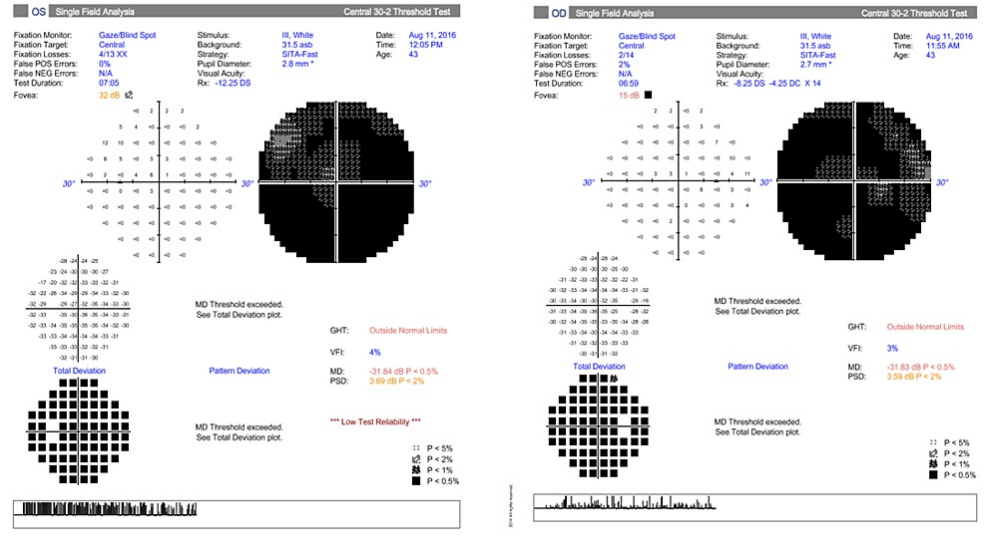
The first patient’s visual field exam (30-2, SITA fast)

The patient was high myopic (-12D in both eyes) without any other ophthalmologic known disease. Our clinical exam revealed a reduced distance best corrected visual acuity (BCVA) (5/10 RE 8/10 LE), impaired color vision in both eyes, as well as a reduced convergence. In fundi, we noticed very small and tilted discs (Figure 1). Her visual field testing showed a severe diffuse concentric deficit in both eyes (Figure 2).

**Figure 3:**
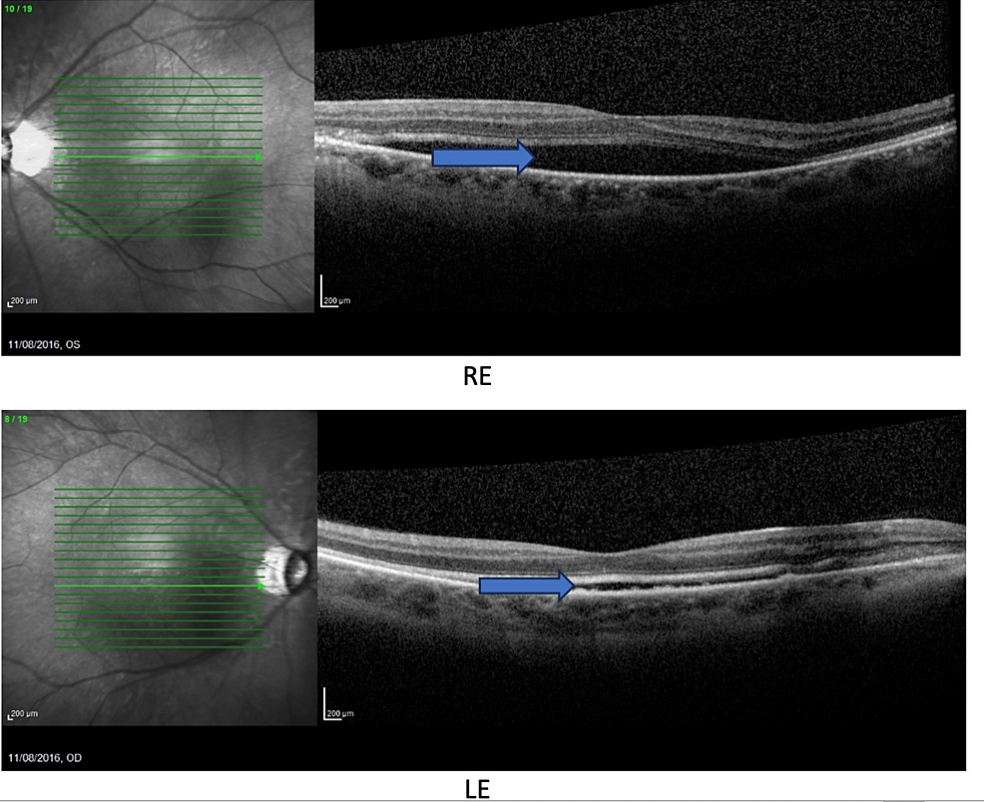
Macular optical coherence tomography RE: right eye, LE: left eye

The macular OCT revealed a bilateral macular retinal serous detachment secondary to pre-eclampsia-related choroidopathy (Figure 3).

**Figure 4:**
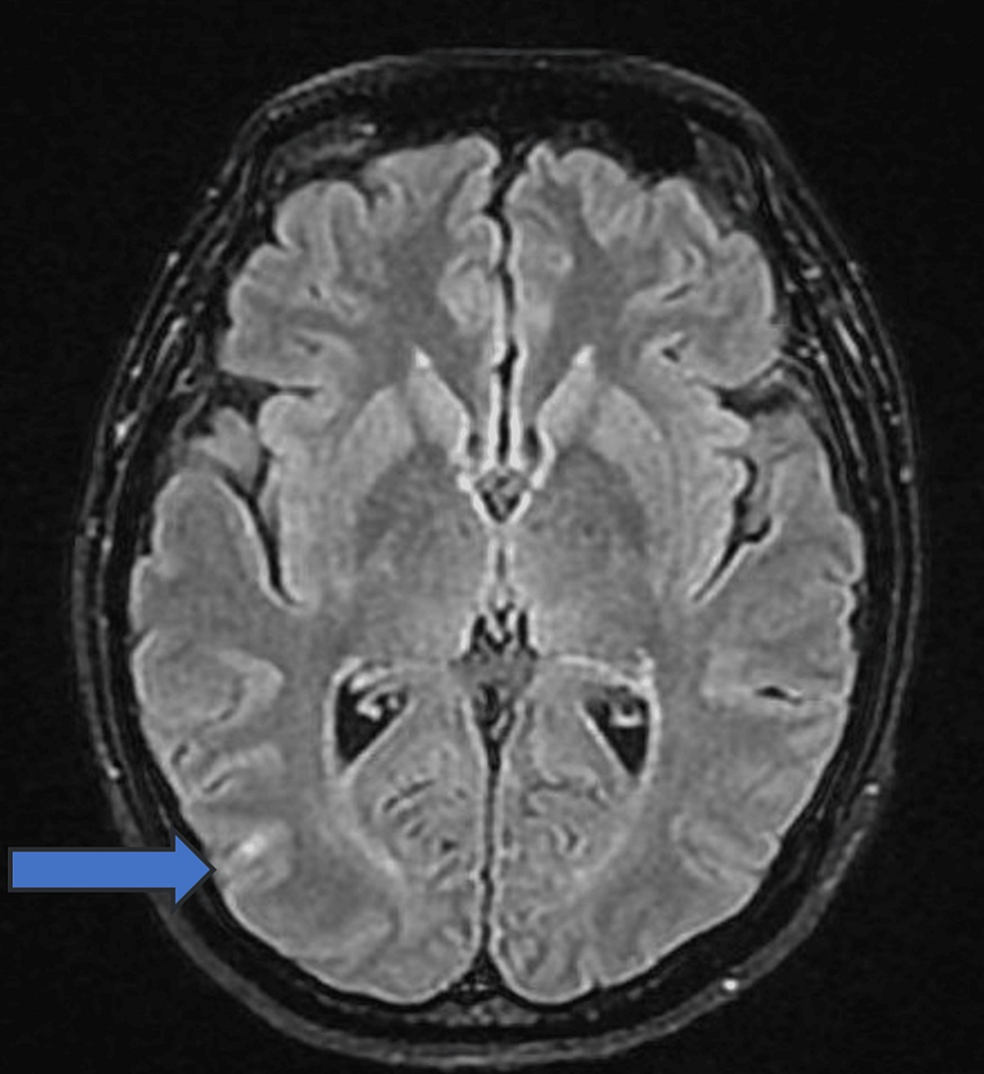
Brain MRI (T2) Note the hypersignal in the temporo-parieto-occipital junction (TPO)

Given this clinical presentation, a brain MRI was conducted to investigate potential neurological causes. MRI showed an acute lesion characterized by an infra-centimetric hypersignal in T2 in the temporo-parieto-occipital junction (Figure 4). In this context, this acute brain damage could result from an acute stroke of arterial origin, a stroke due to thrombophlebitis, or a posterior reversible encephalopathy (PRES) syndrome. While PRES syndrome was considered unlikely, an acute stroke was retained as more possible.

The patient was treated with acetylsalicylic acid 80 mg and Methylopa 250 mg twice a day.

Furthermore, a visual-evoked potential was performed, yielding normal results.

At this stage, the macular retinal serous detachment was not treated, and a wait-and-see attitude was chosen.

**Figure 5:**
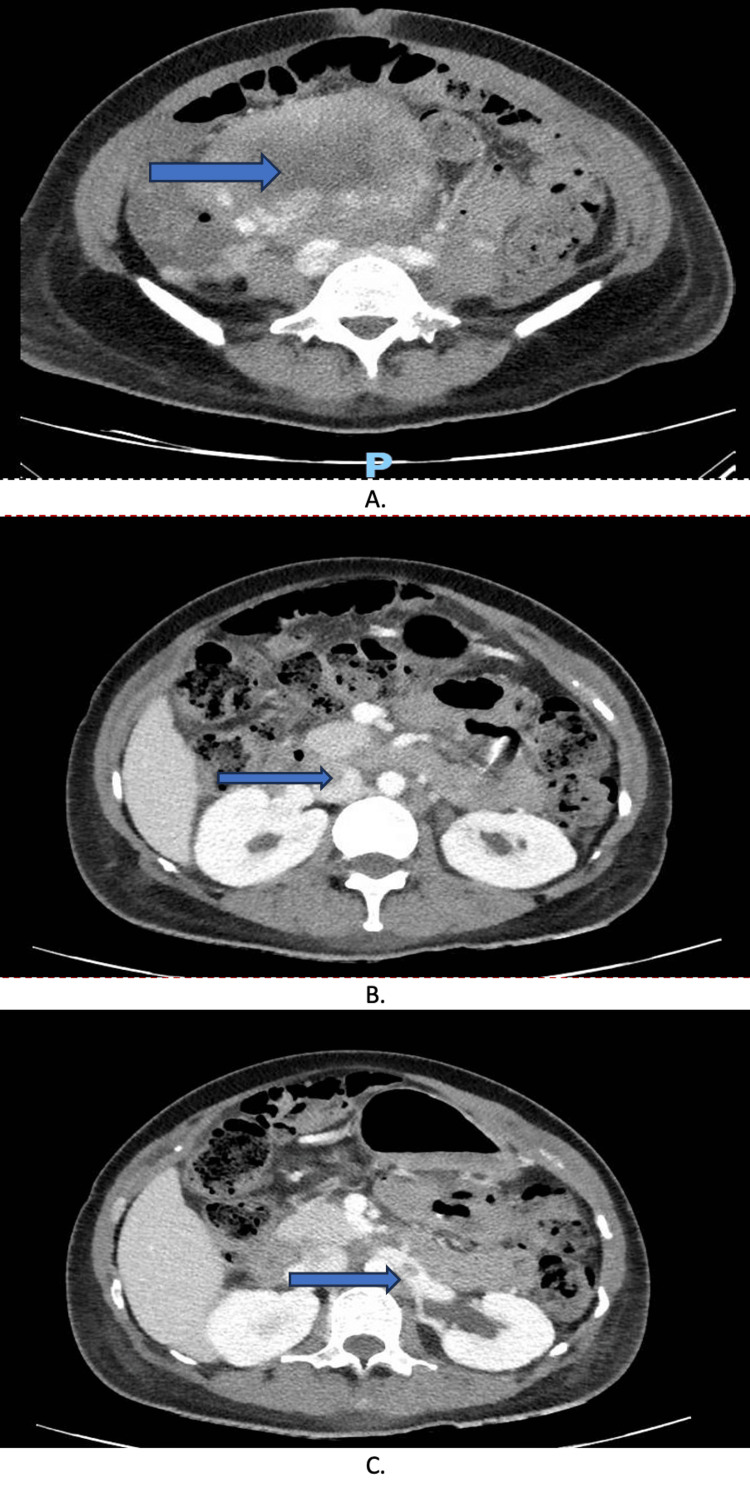
Abdominal CT scan A: Abdominal hematoma, B: Inferior cave vein thrombosis, C: Left renal vein thrombosis

One week later, while the patient was still hospitalized, she experienced significant lower left abdominal pain radiating to the hip. The CT scan divulged an abdominal hematoma along with thrombosis in the inferior vena cava and left renal vein (Figure 5). This was treated by Nadroparin.

At the end of the hospitalization, the patient was evaluated in our ophthalmological department. She continued to report reduced visual acuity and described pain in the right eye.

**Figure 6:**
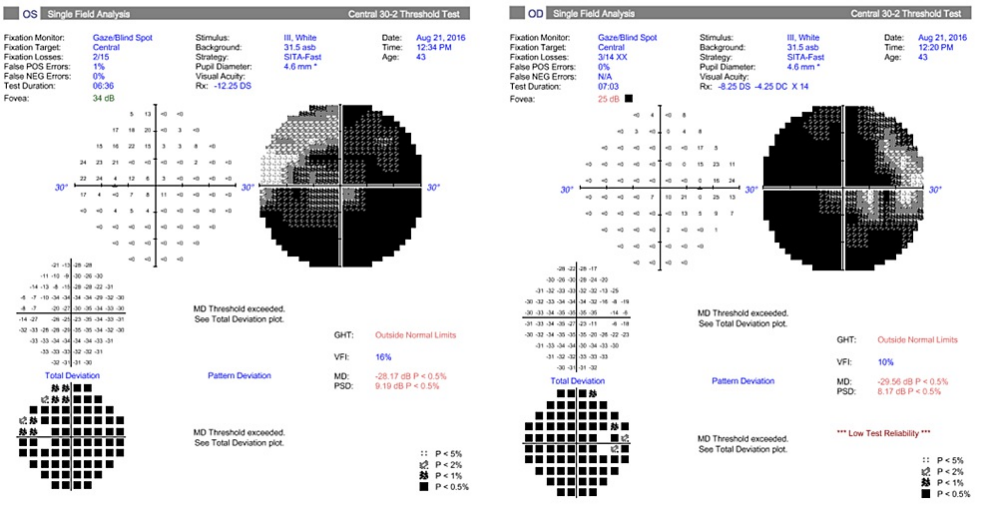
Visual field testing performed 10 days after the first exam

The clinical exam was almost the same as the first time, except for her visual acuity of the left eye, which raised to 10/10. The visual field test showed a slight improvement in the deficit, although it remained significantly impacted (Figure 6).

Since the hospitalization, regular follow-ups have been conducted every couple of months.

**Figure 7:**
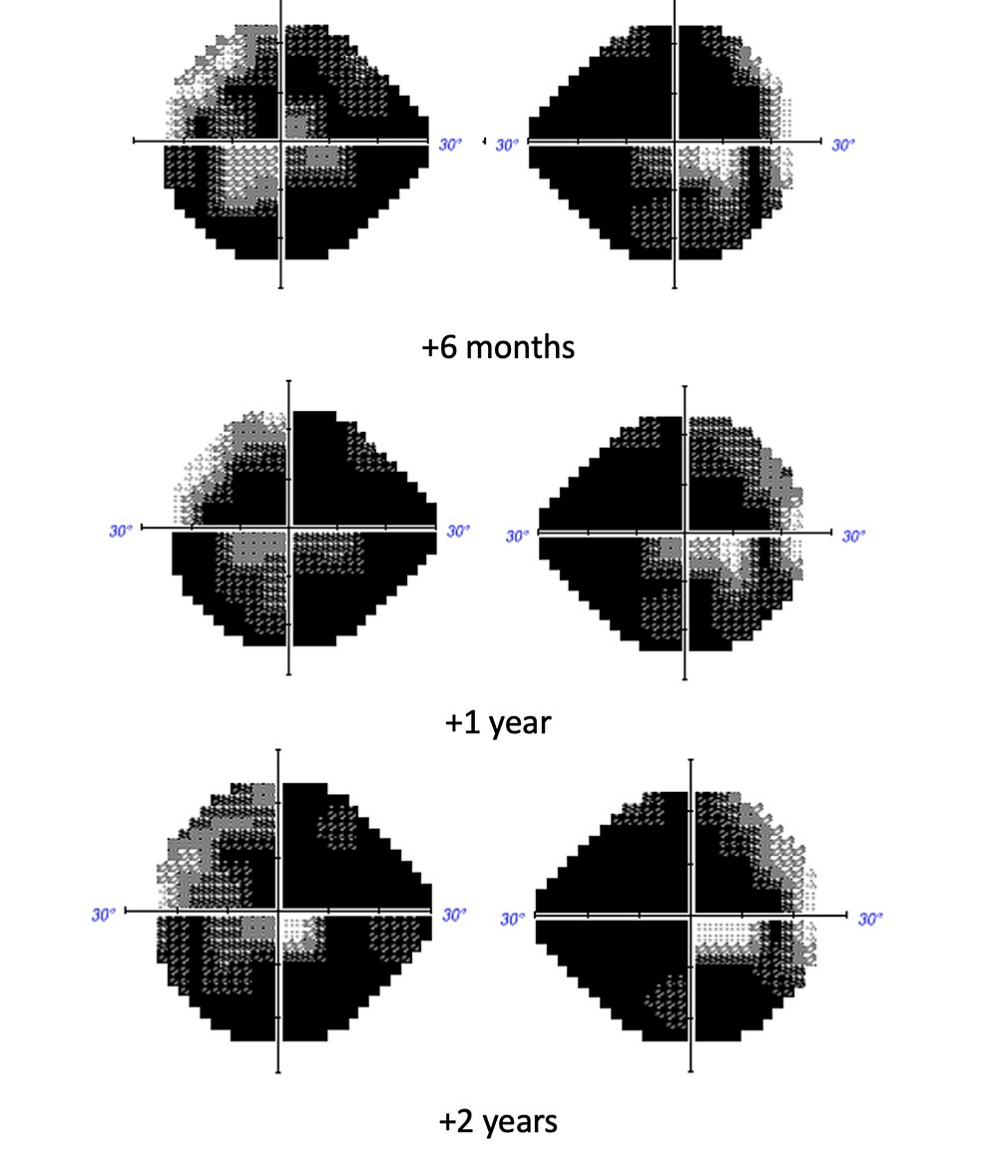
Visual field exams during the follow-up

We have been following the patient for six years; the visual field defect stabilized (Figure 7), and the OCT shows an optic disc and macular atrophy.

However, over the past two years, she experienced a subjective reduction of the visual field as well as the visual acuity without any apparent cause. As a precautionary measure, an MRI was performed, resulting in normal.

Given this clinical progression, a bilateral neuropathy needed to be ruled out. A comprehensive workup, including blood tests, infectious serologies, auto-immune antibodies (anti-NMO/MOG), genetic testing (OPA + Leber), visual evoked potentials (VEP), and a full-field electroretinogram, was conducted. All results were negative, except for the VEP, which indicated delayed conduction in the pre-chiasmatic optic nerves, consistent with optic neuropathy.

**Figure 8:**
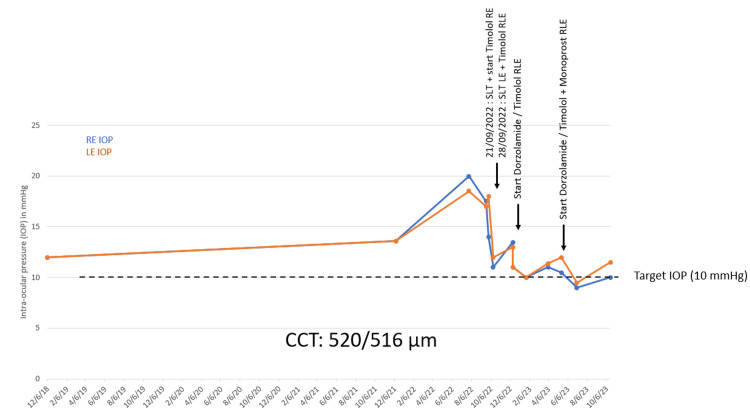
IOP evolution and treatments IOP: Intra ocular pressure, SLT: Selective laser traculoplasty, CCT: Central corneal thickness

As the patient's visual field continued to deteriorate, coupled with her high myopia and low central corneal thickness (520 µm RE and 516 μm LE), there arose a strong clinical suspicion of normal-tension glaucoma. Initial treatment involved selective laser trabeculoplasty in both eyes. Subsequently, local monotherapy with beta-blockers was initiated, followed by bitherapy to attain the target intraocular pressure (Figure 8).

## Discussion

In summary, a 43-year-old female with no prior medical or ophthalmological history, except for high myopia (-12D RLE), experienced an acute subtotal loss of visual field during a twin pregnancy complicated by preeclampsia, HELLP syndrome, and microstroke.

Despite the resolution of her macular choroidopathy, an exhaustive etiological work-up, and optimal management of her “normal-tension glaucoma,” her visual field continues to deteriorate.

This case serves as an illustrative example of the potential interplay of multiple factors leading to a similar clinical manifestation. Undiagnosed glaucoma seems to be the major pathology, as the patient had never consulted an ophthalmologist, and the microstroke and preeclampsia contributed to additional central campimetrical alterations.

The optic nerve is supplied with blood from multiple branches of the ophthalmic artery. This vascularization is susceptible to various pathologies, including arteritic anterior ischemic optic neuropathy (AAION) and non-arteritic anterior ischemic optic neuropathy (NAAION) [3], which are particularly feared. In these conditions, reduced blood flow to the anterior segment of the optic nerve results in permanent structural damage. While ischemic pathology can also affect the posterior segment (posterior ischemic optic neuropathy), such occurrences are very rare. Extensive documentation exists on the diverse local and systemic risk factors associated with these ischemic pathologies.

The impairment of optic nerve blood flow can manifest in a subtle manner, devoid of typical clinical findings or a characteristic patient profile [4]. Such was the case with our patient, who exhibited no signs of optic nerve edema and was a young Asian female without any apparent cardiovascular risk factors.

Numerous explanations may underlie her bilateral optic neuropathy. One possibility is multifactorial optic nerve microvascular damage resulting from high myopia, pre-eclampsia, and blood loss during a cesarean section. While the clinical presentation of acute ischemic neuropathy may not precisely align with the patient's condition, her optic nerve blood flow has probably been affected.

Additionally, the progression of glaucoma, even after achieving target pressure, could contribute to optic neuropathy. Visual field defect progression, in conjunction with intraocular pressure (IOP), is pivotal in assessing disease stability or progression in glaucoma patients. However, in cases where the patient presents with other ophthalmic pathologies, attributing progression solely to glaucoma becomes challenging.

Genetic optic neuropathy remains another potential cause, pending additional genetic testing results. Once again, the typical genetic optic neuropathy profile does not fully correspond with the patient's clinical progression.

Furthermore, the patient has recently been diagnosed with systemic lupus. A literature review suggests that lupus can contribute to visual field defects through microvascular neuropathy or induce glaucoma [5], although the specific mechanisms may not apply to this patient as she did not receive steroids nor exhibit neovascularization [6]. Nevertheless, it is possible that systemic lupus contributed to optic nerve microvascular damage [7,8].

There is a likely interaction among these various possibilities, leading to severe bilateral optic nerve damage.

In such circumstances, it is crucial to preserve the remaining functional optic nerve fibers. To achieve this objective, several ophthalmological and systemic factors must be addressed. Firstly, systemic blood pressure management is paramount, and Ambulatory Blood Pressure Monitoring (ABPM) was conducted to investigate nocturnal hypotension.

Subsequently, achieving target intraocular pressure (IOP) in both eyes is essential. To this end, various therapeutic interventions, including laser treatments and eyedrops, have been employed.

Furthermore, the patient's systemic lupus requires diligent management and monitoring by an internal medicine specialist. Additionally, a lumbar puncture was performed to exclude any signs of neurological inflammatory disease.

Given that the visual field continued to deteriorate, the quality of life of the patient became very impacted. Therefore, she wanted to take all possible measures to slow down the clinical progression as much as possible.

In the literature, numerous cases of pre-eclampsia with visual field defects have been reported, often related to retinal hypertension or PRES syndrome [9]. While these causes typically result in non-permanent visual field defects, that’s not the case for our patient, who presents an unusual outcome. 

To the best of our knowledge, only one similar case involving a severe and persistent concentric visual field defect following pre-eclampsia has been documented in the literature [10]. The case of our patient adds to this body of evidence.

## Conclusions

This clinical case report highlights a complex diagnostic challenge wherein a profound clinical finding lacked a clear and definitive diagnosis. Identifying a precise cause of the patient's visual field defect and devising an effective treatment strategy to halt its progression proved exceedingly difficult. Considering the patient's systemic context, lack of prior ophthalmological examinations, and acute clinical evaluation, a diagnosis of bilateral optic nerve damage involving multiple mechanisms was established.

Very few cases of concentric visual field defect following pre-eclampsia have been documented in the literature. The case of our patient adds to this body of evidence and encourages a deeper exploration of the underlying causes and mechanisms involved. Multidisciplinary management and treatment are very important in complicated cases such as ours.
